# Quantification of hepatic FOXP3+ T‐lymphocytes in HIV/hepatitis C coinfection

**DOI:** 10.1111/jvh.12141

**Published:** 2013-08-15

**Authors:** S. K. Williams, E. Donaldson, T. Van der Kleij, L. Dixon, M. Fisher, J. Tibble, Y. Gilleece, P. Klenerman, A. H. Banham, M. Howard, D. P. Webster

**Affiliations:** ^1^ Brighton and Sussex University HospitalsBrightonUK; ^2^ Brighton and Sussex Medical SchoolBrightonUK; ^3^ Peter Medawar Building for Pathogen ResearchNuffield Department of Clinical MedicineUniversity of OxfordOxfordUK; ^4^ Nuffield Division of Clinical Laboratory SciencesRadcliffe Department of MedicineUniversity of OxfordOxfordUK

**Keywords:** coinfection, FOXP3 protein, hepacivirus, HIV, human, liver, regulatory, T‐lymphocytes

## Abstract

Coinfection with HIV adversely impacts every stage of hepatitis C (HCV) infection. Liver damage in HCV infection results from host antiviral responses rather than direct viral pathogenesis. Despite depressed cellular immunity, coinfected patients show accelerated hepatic fibrosis compared with HCV monoinfected patients. This paradox is poorly understood. T‐regulatory (Treg) cells (CD4+ and FOXP3+) are hypothesized to limit hepatic damage in HCV. Our hypothesis was that reduced frequency of hepatic Treg in HIV/HCV coinfection compared with HCV monoinfection may explain poorer outcomes. We quantified FOXP3+, CD4+, CD8+ and CD20+ cells in liver biopsies of 35 male subjects matched by age and ISHAK fibrosis score, 12 HIV monoinfected, 11 HCV monoinfected and 12 HIV/HCV coinfected. Cell counts were performed using indirect immunohistochemical staining and light microscopy. HIV/HCV coinfected subjects had fewer hepatic FOXP3+ (*P* = 0.031) and CD4+ cells (*P* = 0.001) than HCV monoinfected subjects. Coinfected subjects had more hepatic CD8+ cells compared with HCV monoinfected (*P* = 0.023), and a lower ratio of FOXP3+ to CD8+ cells (0.08 *vs* 0.27, *P* < 0.001). Multivariate analysis showed number of CD4+ cells controlled for differences in number of FOXP3+ cells. Fewer hepatic FOXP3+ and CD4+ cells in HIV/HCV coinfection compared with HCV monoinfection suggests lower Treg activity, driven by an overall loss of CD4+ cells. Higher number of CD8+ cells in HIV/HCV coinfection suggests higher cytotoxic activity. This may explain poorer outcomes in HIV/HCV coinfected patients and suggests a potential mechanism by which highly active antiretroviral therapy may benefit these patients.

**Abbreviations**
FOXP3: forkhead box protein 3
HAART: highly active antiretroviral therapy
HCV: hepatitis C virus
HIV: human immunodeficiency virus

## Introduction

Of 40 million people infected with HIV worldwide, approximately 25–30% are coinfected with hepatitis C virus (HCV) 1. The introduction of highly active antiretroviral therapy (HAART) has significantly improved survival of people living with HIV 3, with HCV‐related liver disease subsequently emerging as a major cause of morbidity and mortality among coinfected patients 4. Therefore, understanding the interactions of these two viruses and the drugs used to treat them is of vital importance.

HIV infection adversely impacts every stage of HCV infection; enhancing HCV transmission, decreasing HCV clearance leading to higher rates of chronic infection 8 and to higher HCV viral loads 9. HIV/HCV coinfected patients have higher necro‐inflammatory activity on liver biopsies and earlier development of end‐stage liver disease 10. The mechanisms underlying this adverse interaction are poorly understood.

Hepatic damage in HCV infection is thought to result from host antiviral responses (including CD4+ and CD8+ T cells) to HCV‐infected hepatocytes rather than direct viral pathogenesis 12. This seems inconsistent with the observation that while HIV/HCV coinfected patients have worse clinical outcomes, they also have an HIV‐mediated depressed cellular immune response 14. Furthermore, data suggest that HAART‐mediated HIV control limits liver damage in HIV/HCV coinfection 16. This paradox has, as yet, not been fully explained.

CD4+ T‐regulatory cells (Treg) play an important role in self‐tolerance and down‐regulation of immune response, having been demonstrated to decrease autoimmune activity 17. Forkhead box Protein 3 (FOXP3) is a transcriptional factor essential to Treg function 18 and has been used as a marker of Treg activity 19. In HCV infection, Tregs have been associated with impairment of peripheral blood immune responses and associated with HCV chronicity 21. HCV patients have higher levels of Treg suppressive activity in blood compared to healthy controls 22. *Ex vivo* studies on hepatic tissue demonstrate that CD4+ FOXP3+ cells are the major intrahepatic Treg population in HCV‐infected patients 24. Higher FOXP3 activity is inversely proportional to hepatic fibrosis in chronically infected patients, and so these cells have been suggested as protective 24.

Several studies describe changes in the frequency of circulating Treg in HIV infection, increasing as a relative proportion of CD4+ T cells, but declining as CD4+ T‐cell count declines 25. There are conflicting data on circulating Treg populations in individuals infected with HCV 25 and also in studies comparing HCV monoinfection with HIV/HCV coinfection 25. However, there are no data on these regulatory cells directly from the liver in HIV/HCV coinfection. As the liver is the primary site of pathology and the area where Treg are substantially enriched in HCV monoinfection, this is an important question in understanding the enhanced pathology in HIV/HCV disease. Our primary hypothesis was that reduced frequency of hepatic FOXP3+ Treg in subjects with HIV/HCV coinfection compared with HCV monoinfection may explain the poorer clinical and histological outcome in the coinfected group. We therefore analysed liver tissue from HCV monoinfected, HIV/HCV coinfected and HIV monoinfected subjects and quantified FOXP3+ T cells in relation to infection status.

## Materials and Methods

### Ethics

The protocol conforms to ethical guidelines of the Declaration of Helsinki (1975), and ethical approval was granted by the Research and Development Department of Brighton and Sussex University Hospitals NHS Trust and the National Research Ethics Committee (NREC). Data were held in accordance with the Data Protection Act (1998) and NHS Venereal Diseases Regulations (1974).

### Liver tissue

Formalin‐fixed, paraffin‐embedded liver biopsies from patients seen at the Royal Sussex County Hospital, Brighton, UK, were obtained from Pathology Department archives. These were categorized as having HIV/HCV coinfection, HCV monoinfection or HIV monoinfection, and 12 patients were selected from each category. Patients were matched in terms of age (±7 years) and fibrosis score and scaled to represent a range of disease stages from very mild fibrosis to cirrhosis. Due to the nature of the population demographic, all subjects were male and non‐African. Exclusion criteria were age under 18 years of age, HIV‐2 infection and hepatitis B coinfection. The rationale for liver biopsy in patients with HCV monoinfection and HIV/HCV coinfection was staging of their liver fibrosis in relation to hepatitis C infection. Patients in the HIV monoinfection group were biopsied for range of non‐infective, non‐autoimmune reasons, including staging of fibrosis in relation to clinical suspicion of alcoholic liver disease, non‐alcoholic fatty liver disease and nevirapine‐induced liver toxicity.

### Clinical data

Clinical and demographic data were obtained from hospital notes and hospital Pathology and Clinical Biochemistry databases.

### Immunostaining and cell counts

We modified a protocol initially described by Ward *et al*. 24. All procedures were performed by investigators blinded to the classification of the subjects. First, six serial sections 1.5 μm thick were cut and mounted onto electrically charged slides (Snowcoat X‐tra; Surgipath, Eagle River, WI, USA) before oven‐baking for an hour. Next, they were pretreated at 90 °C in alkali solution for 20 min to achieve antigen‐retrieval postfixation. Envision FLEX Peroxidase‐blocking reagent (DakoCytomation, Glostrup, Denmark) was then applied for 5 min to block endogenous peroxidase, preventing non‐specific staining, followed by a buffer rinse.

Primary antibody [mouse anti‐human CD4, CD8, CD20 (Dako, Glostrup, Denmark) or FOXP3 (‘in house’ hybridoma supernatant, clone 236A/E7)] was then applied to sections for 20 min, followed by buffer rinse. Secondary anti‐mouse antibody (horseradish peroxidase; DakoCytomation) was then applied and incubated for 20 min to achieve signal amplification and to improve antigen detection, followed by buffer rinse. DAB chromagen substrate‐working solution (DakoCytomation) was then applied for 10 min to stain bound antibody brown and then rinsed. This was followed by application of haematoxylin (DakoCytomation) for 5 min, rinsing in water, buffer and finally water again. All sections were stained in parallel with a negative control which omitted the primary antibody step to ensure the specificity of the antibody. The staining procedure was standardized by staining slides on a Dako automated staining machine using an EnVision (Dako) staining system.

Cell counts of portal tracts were performed on serial sections cut from the same block of liver tissue by two separate observers blind to the clinical status of the patient. Stage of fibrosis was recorded on original collection of tissue by Pathology Services at Brighton and Sussex University Hospitals, according to original criteria set by Ishak *et al*. 30.

### Statistical analysis

Analysis was performed using SPSS version 20 (IBM, New York, NY, USA). One‐way ANOVA testing was used to detect differences in cell count across disease groups, with *post hoc* Tukey B testing used to detect significance between the disease groups. Multivariate logistic regression analysis was used to test interdependence of number of FOXP3+ cells and CD4+ cells predicting disease status. One participant was removed as an outlier, with staining for all cell types far exceeding other participants, and with notably high ISHAK score and raised serum transaminases at time of biopsy.

## Results

### Demographics

The available clinical characteristics of each study participant at time of biopsy are summarized in Table 1. Due to the retrospective nature of the study, we were unable to locate some data. One‐way ANOVA testing demonstrated that there was no significant difference between disease groups for age, fibrosis score, antiretroviral medication use in context of HIV infection, blood CD4 count or liver function tests (Table 2). Two patients in the HIV‐only group were on atazanavir at time of biopsy, which is known to cause an isolated rise in bilirubin; however, they had serum bilirubin levels within the normal range. Of those participants infected with HIV, the majority had undetectable viral loads at the time of biopsy, with over 90% of them being on HAART. Of those infected with HCV, the majority did not have HCV viral load quantified at the time of biopsy.

**Table 1:** Clinical data of study participants at time of biopsy

Age (years)	Status	Fibrosis (ISHAK score/6)	Blood CD4 absolute (×10^6^/L)	Blood CD4 (%)	Albumin (g/L)	Total bilirubin (g/L)	ALP (IU/L)	ALT (IU/L)	HCV genotype	ARV treatment
40	HIV/HCV	2	783	28	47	5	120	136	1a	Yes
42	HIV/HCV	0	416	23	46	8	99	76	4d	Yes
43	HIV/HCV	2	552	26	44	6	121	298	4	Yes
46	HIV/HCV	1	729	39	44	6	67	77	–	Yes
44	HIV/HCV	1	264	16	48	8	148	205	2b	Yes
55	HIV/HCV	2	462	22	43	8	77	69	1a	Yes
47	HIV/HCV	2	240	26	46	7	73	103	1a	Yes
56	HIV/HCV	1	712	35	45	10	68	71	1a	Yes
53	HIV/HCV	2	231	20	43	8	59	222	1	Yes
36	HIV/HCV	4	–	–	46	8	68	32	1a	No
38	HIV/HCV	2	–	–	47	2	103	58	–	Yes
57	HIV/HCV	2	574	14	42	8	76	29	–	Yes
38	HCV‐only	1	–	–	45	14	74	145	–	–
45	HCV‐only	0	–	–	50	6	73	33	–	–
38	HCV‐only	1	–	–	47	9	76	85	1	–
50	HCV‐only	0	–	–	45	4	82	23	1a	–
42	HCV‐only	1	–	–	44	20	83	79	–	–
53	HCV‐only	2	–	–	45	9	74	47	1a	–
53	HCV‐only	3	–	–	50	14	150	64	3a	–
54	HCV‐only	2	–	–	43	9	100	83	1a	–
55	HCV‐only	2	–	–	43	7	74	53	–	–
34	HCV‐only	5	–	–	47	36	63	51	1a	–
45	HCV‐only	6	–	–	45	9	79	72	3a	–
38	HIV‐only	1	305	15	46	3	106	93	–	Yes
44	HIV‐only	0	649	26	48	18	59	51	–	Yes
47	HIV‐only	0	363	30	49	9	28	38	–	Yes
48	HIV‐only	0	908	44	51	4	107	173	–	Yes
39	HIV‐only	0	697	36	47	64	120	72	–	Yes
53	HIV‐only	2	645	22	47	9	220	22	–	Yes
48	HIV‐only	0	742	25	48	9	98	76	–	Yes
50	HIV‐only	0	761	20	46	25	96	43	–	Yes
53	HIV‐only	0	481	31	44	11	161	133	–	Yes
41	HIV‐only	3	659	31	44	12	55	127	–	Yes
47	HIV‐only	3	946	36	45	7	97	79	–	Yes
61	HIV‐only	3	457	19	44	25	122	105	–	No

ALP, alkaline phosphatase; ALT, alanine aminotransferase; ARV, antiretroviral medication.

**Table 2:** Summary of clinical parameters by disease group. Mean values in each disease group – mean age, fibrosis score, CD4 and liver function tests

Variable (mean)	HIV/HCV	HCV‐only	HIV‐only	Significance
Age (years)	46	46	47	0.90
ISHAK score (/6)	1.8	2.1	1.0	0.19
Total bilirubin (g/L)	7	12	16	0.13
ALT (IU/L)	115	67	84	0.16
ALP (IU/L)	90	84	106	0.35
Blood CD4 (×10^6^/L)	496	–	634	0.40

### FOXP3 staining cells in patients with HCV, HIV/HCV coinfection and HIV

**Figure 1:**
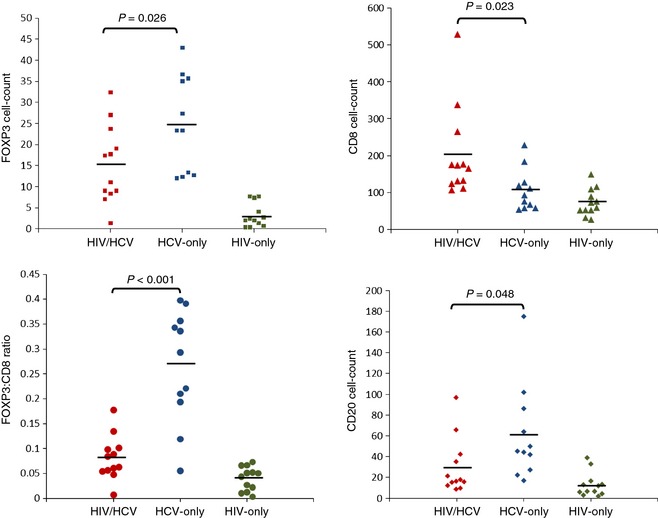
Mean counts of each cell type by disease group. FOXP3+ (top left), CD8+ (top right), FOXP3/CD8 ratio (bottom left) and CD20+ staining cells (bottom right) in portal tracts divided by disease group. Significance values for difference between HIV/HCV and HCV‐only groups (ANOVA) are shown with a *P*‐value. Horizontal bars represent mean values.

**Figure 2:**
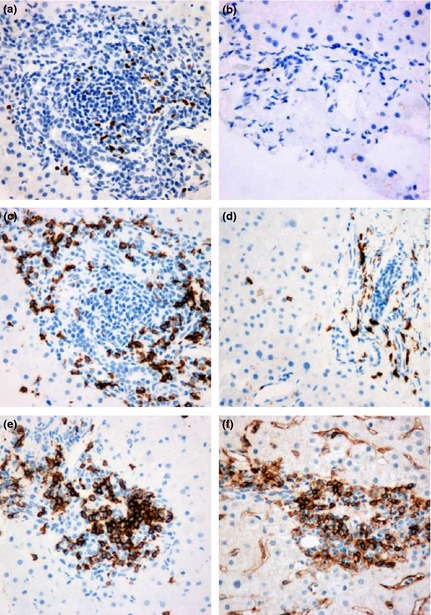
Single immunostaining (brown stain) in serial sections of portal tract liver tissue. High FOXP3 staining in a HCV‐only patient (a) compared with lower staining in a HIV/HCV patient (b). High CD8 staining in a HIV/HCV patient (c) compared with lower staining in a HCV‐only patient (d). Staining for (e) CD4 and (f) CD20. Photographs at 40× magnification.

Our primary intention in this study was to quantify FOXP3+ cells in portal tract tissue and in particular to look for differences in patients with HIV/HCV coinfection compared to those with HCV monoinfection. We observed that there were significantly fewer FOXP3 staining cells in the portal tracts of patients with HIV/HCV coinfection when compared with those with HCV monoinfection (*P* = 0.026). In turn, there were significantly fewer in the HIV‐only group compared to either of the HCV groups (*P* < 0.001) (Figs 1 & 2; Table 3).

**Table 3:** Mean cell counts in portal tracts by disease group

Mean	HIV/HCV	HCV‐only	HIV‐only
FOXP3+	15	25	3
CD4+	38	93	24
CD8+	203	107	74
CD20+	30	61	13

### Relationship between disease group, FOXP3 staining cells and CD4 staining cells

As FOXP3 cells are a subset of CD4 cells, we analysed the number of CD4+ cells in portal tracts. Serial sections were stained for CD4 and FOXP3 in turn. We observed that the mean number of CD4+ cells in portal tracts in each disease group followed the same order of magnitude as FOXP3+ cells. The HCV‐only group had significantly more CD4+ cells than either the HIV/HCV or HIV groups (*P* < 0.001). The difference between either HIV group was not significant (*P* = 0.50).

In order to assess to what extent the observed differences in frequency of FOXP3 cells in portal tracts were related to overall changes in frequency of CD4 cells, we examined the FOXP3/CD4 ratio in each disease group and then performed multivariate analysis including both variables. We observed that there was no significant difference in FOXP3/CD4 between HCV and HIV/HCV groups (*P* = 0.12).

On univariate logistic regression both hepatic FOXP3 (*P* = 0.05) and hepatic CD4 cells (*P* = 0.02) predicted disease status of either HCV monoinfection or HIV/HCV coinfection. However, on multivariate analysis including both variables, FOXP3 no longer independently predicted disease status (*P* = 0.817), whereas CD4 remained significant (*P* = 0.036).

In summary, there is a significant difference in the number of portal tract cells expressing FOXP3 between the HCV and HIV/HCV groups (and indeed the HIV‐only group). This difference is largely driven by the substantial differences in CD4+ T cells within the tissue.

### Relationship between disease group, FOXP3 staining cells and CD8 staining cells

There were significantly more CD8+ T cells in portal tracts of the HIV/HCV group than either the HCV group (*P* = 0.023) or HIV‐only group (*P* = 0.001) (Figs 1 & 2). There was no significant difference in the number of CD8+ cells between the HCV and HIV‐only groups (*P* = 0.6).

As CD8+ cells are thought to be responsible for hepatic cytotoxicity in patients with HCV, and FOXP3+ cells responsible for immunoregulation, we examined the FOXP3 to CD8 ratio, as an indicator for the degree of immune control. There was a striking difference between the HCV‐only and HIV/HCV groups, with a significantly higher FOXP3/CD8 ratio in the HCV‐only group in comparison with the HIV/HCV group (0.27 *vs* 0.08, *P* < 0.001).

Across disease groups, we found a positive linear relationship between FOXP3+ and CD8+ cells (*r*^2^ = 0.16, *P* = 0.018). Analysing this association within disease groups revealed a strong positive linear association within the HIV/HCV (*r*^2^ = 0.468, *P* = 0.014) and HIV‐only (*r*^2^ = 0.663, *P* = 0.001) groups and a weakly positive but not significant relationship in the HCV‐only group (*r*^2^ = 0.024, *P* = 0.652).

Essentially, we observed that the expression of CD8+ and FOXP3+ cells is linked and that where there are more CD8+ cells, there is a reflex increase in FOXP3+ cells. However, in the HIV/HCV coinfected group, the overall number of CD8+ cells is much higher and the ratio of FOXP3+ to CD8+ cells is much lower when compared with the HCV monoinfected group. This reflects that within groups you see a reflex increase in FOXP3+ cells as CD8+ cells increase; however, this effect is blunted within the coinfected group when compared with the HCV monoinfected group.

### Relationship between disease group and CD20 staining

The mean number of CD20+ cells per portal tract was significantly higher in the HCV‐only group compared with the HIV/HCV group (*P* = 0.048). There were fewer CD20+ cells in the HIV‐only group, a difference that was significant compared with the HCV‐only group (*P* = 0.002) but not compared with the HIV/HCV group (*P* = 0.39).

## Discussion

The primary finding in our study is that the number of hepatic FOXP3+ cells per portal tract was significantly lower in HIV/HCV coinfected patients compared with HCV monoinfected patients. As we have previously shown that the vast majority of FOXP3+ T cells in these infiltrates are CD4+ T cells, this indicates lower number of Treg in coinfection and therefore this population may exhibit a lower overall level of regulatory activity. This finding could account for the worse histological and clinical outcome of these patients. Furthermore, the observation that lower number of FOXP3+ cells is accompanied by proportionately lower number of CD4+ cells in the HIV/HCV coinfected group suggests that lower number of FOXP3+ cells (and therefore Treg) probably reflects the lower overall number of hepatic CD4+ cells rather than a specific loss of Treg. The observation of significantly lower number of both CD4+ and FOXP3+ cells in the HIV‐only group compared with either of the HCV groups reflects both their immunodeficiency and absence of a hepatic pathogen. This is consistent with previous studies which have demonstrated that a higher number of FOXP3+ cells are present in livers of HCV‐infected patients, and that FOXP3+ Treg play an important role in this condition, compared with other causes of liver fibrosis 24. The CD4 counts of our coinfected cohort were relatively well preserved (mean 496) with 11 of 12 subjects being on HAART. This suggests that the loss of regulatory activity within the liver occurs well before significant immunocompromise and at near normal blood CD4 counts.

The next important finding is that the HIV/HCV coinfected group had significantly higher number of hepatic CD8+ cells compared with the HCV‐only group, and the ratio of FOXP3+ to CD8+ cells was significantly lower in the coinfected group. As CD8+ cells exhibit cytotoxic activity and are thought to be responsible for accelerated fibrosis in HCV infection 31, this may explain the worse clinical outcome in the coinfected group. Furthermore, as FOXP3 (Treg) is thought to down‐regulate cytotoxic CD8+ T‐cell activity, the finding that the HIV/HCV group has a lower FOXP3/CD8 ratio to a high degree of significance suggests that this group have a lower degree of immune control compared with the HCV‐only group. Furthermore, we observed a positive correlation between CD8+ and FOXP3+ cells across disease groups, providing evidence that expression of these cell types are closely interlinked. It suggests that immunoregulatory intrahepatic FOXP3+ cells increase proportionately in response to higher number of cytotoxic CD8+ cells, albeit in smaller numbers in immunodeficient coinfected patients.

Finally, our study demonstrates that the HIV/HCV coinfected group had significantly fewer hepatic CD20+ cells than the HCV monoinfected group. As CD20 is a marker for B cells, this finding suggests impaired humoral immunity in the coinfected group. Furthermore, B cells have been shown to play a regulatory role although the nature of this in HCV has not been established 32. This provides evidence of alternate mechanisms in which HIV infection may impair immunity in the context of HCV and thus lead to poorer outcomes in coinfected patients.

This is a preliminary retrospective study and as such is limited by the number and types of assays that could be performed. Using immunohistochemistry on archived liver tissue, we have shown the inter‐relatedness of FOXP3+ and CD8+ cells and their importance in HIV/HCV coinfection. Further studies including flow cytometry are warranted on fresh liver samples with concomitant assays on peripheral blood mononuclear cells. While FOXP3 remains a preferred marker for Treg, the isolation of these cells in human tissue remains a challenge due to the possibility of heterogeneity in surface proteins 33. Other authors have suggested CD4+ CD25+ cells that have low CD127 expression as an alternate marker; however, further studies are required before deciding on a definitive marker for Treg 34. Furthermore, the liver tissue available in our histology archive limited the spectrum of disease that could be studied. While we have matched samples from HIV/HCV coinfected individuals and HCV monoinfected cases by fibrosis score (as well as age and gender), these findings need to be replicated in a larger, prospective, multicentre study that would also include female participants and different ethnicities in order for these results to be generalisable.

In conclusion, our study provides novel data on the balance between intrahepatic T‐cell populations in patients coinfected with HIV and HCV. The worse clinical and histological outcome in coinfected patients despite their immunodeficiency has long been at odds with dominant understanding that hepatic damage is a consequence of immune overactivity. Due to the observational nature and temporal limitations of our study, we are unable to attribute causality; however, our data supports a theory that in the context of HCV infection, HIV infection decreases number of FOXP3+ CD4+ Treg cells, leading to decreased immunoregulation and increased pathology due to cytotoxic CD8+ T‐cell populations. Early HAART is recommended in patients with coinfection, and this study provides a plausible mechanism as to how this might provide benefit, that is if HAART can be started before CD4 cell numbers decline, then Treg numbers and immune regulatory activity may be preserved. The fact that liver regulatory activity appears to be depressed in coinfected patients, even with relatively well‐preserved CD4 counts, suggests that very early HAART may benefit this group. This would therefore support the rationale for initiating HAART earlier in patients with HIV/HCV coinfection. This could serve to reduce HCV disease progression though in this retrospective study we are not able to demonstrate this. It would have been interesting to study patients with lower CD4 counts and those not on HAART. However, in the modern antiretroviral era, there are few of these patients in cohorts, particularly given the trend to start early HAART in hepatitis virus coinfected patients. Finally, our data suggest that in this coinfected population, HIV infection also has a detrimental effect on number of CD20+ cells, thus potentially weakening local humoral immunity. We believe our findings may explain in part why coinfected patients have a worse overall outcome and propose that future prospective studies of FOXP3 cells in these populations may be of value in defining disease progression.

## Conflicts of Interest

AH Banham receives royalty payments from the licensing of the 236A/E7 FOXP3 monoclonal antibody.
